# Powder Fever and Its Impact on Decision-Making in Avalanche Terrain

**DOI:** 10.3390/ijerph18189496

**Published:** 2021-09-09

**Authors:** Andrea Mannberg, Jordy Hendrikx, Jerry Johnson, Audun Hetland

**Affiliations:** 1 School of Business and Economics, Faculty of Biosciences, Fisheries and Economics, UiT the Arctic University of Norway, 9037 Tromsø, Norway; andrea.mannberg@uit.no; 2 Snow and Avalanche Lab, Department of Earth Sciences, Montana State University, Bozeman, MT 59717, USA; jordy.hendrikx@montana.edu; 3 Department for Political Science, Montana State University, Bozeman, MT 59717, USA; jdj@montana.edu; 4 Department of Psychology, Faculty of Health Sciences, UiT the Arctic University of Norway, 9037 Tromsø, Norway

**Keywords:** affect, arousal, decision-making, risk assessment, risk-taking

## Abstract

We examined the effect of emotions, associated with “powder fever”, on decision-making in avalanche terrain. Background: Skiing in avalanche terrain is a voluntary activity that exposes the participant to potentially fatal risk. Impaired decision-making in this context can therefore have devastating results, often with limited prior corrective feedback and learning opportunities. Previous research has suggested that arousal caused by emotions affects risk assessment and intentions to engage in risky behavior. We propose that powder fever may induce similar responses. Methods: We used the following two experimental methods: laboratory studies with visual visceral stimuli (ski movies) and a field study with real stimuli (skiing exciting terrain). We evaluated the effect of emotions on attention, risk assessment, and willingness to expose oneself and others to risk. Results: Both the laboratory studies and the field study showed that skiing-related stimuli had a relatively strong effect on reported emotions. However, we found very few significant effects on decision-making or assessment of risk. Conclusions: Skiing activities make people happier. However, despite the clear parallels to sexual arousal, powder fever does not appear to significantly impair decision-making in our study. More research on the effects of powder fewer on milder forms of risk-taking behavior is needed.

## 1. Introduction

Backcountry skiing, known as off-piste in Europe, is a rapidly growing leisure activity [1,2]. People who engage in backcountry skiing either ride in potential avalanche terrain that is in near proximity to ski areas but not controlled by the ski patrol [3,4], or in remote mountainous areas. Like many other outdoor sports, backcountry skiing is associated with risks. In the backcountry, riders face the risk of avalanches in addition to other risks, e.g., injuries caused by a fall or by severe weather. Each year, an average of 100 people in the European Alps, and 40 people in North America die in avalanche accidents [5,6,7]. This does not include near miss accidents where injury rather than death was the result. In 90% of all fatal avalanche accidents, the victim or a member of the victim’s group is the triggering mechanism for an avalanche [8]; therefore, the decisions made by an individual or group play a crucial role in safe ski touring practice.

Research has demonstrated that people experiencing strong emotions (a so-called hot state), such as sexual arousal [9] or anger [10], express higher risk-taking propensity than people in a neutral state. Ariely and Lowenstein [9] showed that sexual arousal had a strong impact on judgment and decision making related to sex. Rydell et al. [10] examined intergroup anger and found that anger reduced the systematic processing of persuasive messages and, when compared to intergroup fear, increased risk taking. These two studies suggest that hot states may impair judgments and decision-making.

In this paper, we tested if emotions associated with “powder fever” affect skiers’ attention to danger signs, risk perception, and willingness to expose themselves and others to risk. Powder fever is a concept used by riders (skiers, snowmobilers, etc.) to describe the euphoric feeling and excitement, or anticipation that arises when they either ride or anticipate riding fresh snow or “powder”. Given the impact that sexual arousal and arousal invoked from anger have been demonstrated to have on decision making, it seems plausible that powder fever may also influence decision making in this specific setting. A better understanding of the link between the emotions associated with powder fever and decisions in avalanche terrain hold potential to improve our understanding of avalanche accidents and lower the costs of poor decisions. The reduction in avalanche accidents and injury has clear benefits to the immediate skiing community but also for others who may be endangered by the unsafe practices of others (i.e., causing an avalanche to release on a skier’s downslope), causing search and rescue resources to be deployed, and endangering others in an avalanche rescue scenario [11]. In addition, knowledge on the link between emotions and risk assessment in the backcountry could improve our understanding of excessive risk taking in other leisure activities. This is especially important during pandemic conditions when medical resources are stretched thin and volunteer responders are potentially exposed to COVID-19.

### 1.1. Emotions

Emotions influence literally all aspects of human functioning such as attention, inference, learning, memory, physiology, self-concept, goal choice, perception, and decision making [12,13]. The very function of our emotions is to assist us in adapting to the environment by guiding our attention and prepares us to act according to a goal. However, since emotions focus our attention on one goal, they can detract from competing goal pursuits [14,15]. Although our emotional reactions depend on both dispositional and situational factors, many theorists claim that the emotional system can be organized into two distinct motivational subsystems; pleasant affect and unpleasant affect [16,17,18,19]. Several studies report that experiencing positive emotions is among the key motivational factors for taking part in backcountry skiing, or other challenging activities [20,21,22].

Different emotions activate a predefined set of cognitive “checklists” and primes us for a set of actions [14]. Emotions thus save cognitive processing by triggering what Levenson [23] calls time-tested responses to universal experiences such as loss, sexual attraction, or a threat. In the case of a threat, the feeling of fear sets up our cognitive and endocrine systems to confront the danger and puts our body in a fight or flight mode. When we experience fear, we produce adrenaline, our heart rate and blood pressure increase, and our facial expression, vocal pitch, and body posture change [24,25,26,27,28]. In terms of action readiness, all ongoing activity terminates, and our focus is solely directed to the source of danger, preparing us to execute appropriate counter actions. On the positive side, happiness is a reward for reaching a goal or making progress on a plan [14].

#### 1.1.1. Hedonic and Eudaimonic Emotions

The distinction between making progress and reaching a goal is significant. Several scholars make a distinction between hedonic emotions such as pleasure, satisfaction, and happiness and eudaimonic emotions such as interest, enthusiasm, and engagement [29,30]. From a physiological point of view, the function of hedonic emotions such as pleasure is to signal to the body that a return to a homeostatic set point has been achieved, such as eating when hungry or becoming warm after being cold [30]. From a psychological point of view, the function of hedonic emotions is to signal that a goal has successfully been reached [31]. Eudaimonic emotions such as interest, enthusiasm, and engagement, in contrast, serve as motivational signals that propel one toward a goal. This is elegantly summarized in a paper from Barbano and Cador [32] titled: “Opioids for hedonic experiences and dopamine to get ready for it”. The authors claim that dopamine seems more important in approach or “wanting” behavior, whereas hedonic emotions seem regulated by other brain systems responsible for reward, tied to endogenous opioids. Even though the two kinds of positive emotions serve different functions, they are both positive [31]. Powder fever likely has elements of anticipation and reward, we therefore include both classes of positive emotions in our study. 

Emotions also guide the magnitude and direction of our depth of thought or deliberate reasoning. In general, a negative situation will lead to the deployment of more deliberate resources while a positive situation needs fewer deliberate resources. However, there are differences between the two classes of positive emotions. Eudaimonic emotions like interest motivates focused attention over time [33] whereas happiness leads to a broadening of attention [34]. Decision-heuristics are simple rules of thumb, which reduces the effort of making a decision to arrive at satisfactory outcomes/solutions [35]. The use of heuristics does not necessarily lead to poor decisions. Numerous studies have shown that simple heuristics match or even outperform more complex decision algorithms in several fields such as medicine, finance, management, and law [36,37,38,39]. However, heuristics necessarily trade off some accuracy for less effort [35]. Emotions affect our tendency to rely on heuristics when we make decisions [36,37]. For example, happy people appear to be more prone to use heuristics than fearful people [13]. Angry individuals perceive negative events to be under human control, and brought about by others, whereas people that are afraid perceive the negative events to be unpredictable and under situational control. Hence, emotions that signal high control, such as anger or happiness, will result in substantially lower risk perception compared to emotions that signal lower levels of control such as fear [38].

#### 1.1.2. The Link between Emotions and Risk-Taking in Sporting Activities

Engagement in sporting activities give rise to a range of emotions that hold power to affect risk estimates, judgements, and ultimately decisions to take risks. Several mechanisms create these links.

First, when we engage in sport, we often compete either with others, or with ourselves. Competition gives rise to the fear of losing and has been shown to increase willingness to take risks [39,40]. Second, physical activity gives rise to arousal, e.g., via an increase in the heart rate. Previous research has shown that activity-induced arousal reduces risk estimates and risk judgments [39,40], and can increase risk-taking [41]. Black et al. [41] demonstrated that people who first engaged in a competitive exercise (playing tennis), took more risk in an unrelated task immediately following the exercise.

Black et al. [41] proposed the following two explanations for their findings: (1) the increase in the level of dopamine from the intense exercise leads to a search for further stimulation through risk-taking, and (2) the tiredness from the intensive exercise induces performance errors. The second explanation has been supported by research, which showed that the tiredness resulting from physical activity limits cognitive abilities [39,42].

Third, endorphins released during physical exercise affects emotions (primarily a reduction in anxiety) and mood states [43]. A mood state is a psychological condition that is typically of longer duration, more general, and less intense than an emotional state. Mood states directly influence risk perception and risk-taking behavior. Negative mood states are associated with increased risk-taking tendencies, whereas positive mood states are associated with lower risk taking [44,45,46].

Fourth, challenging physical activities can produce a pleasant state known as “flow”. The sense of “flow” may occur when the challenge match or slightly exceed our level of skill [47,48] i.e., when we are just barely in control. Feelings of control reduce the perceived risk [37,38]. Delle Fave et al. [46] noted that the opportunity to experience “flow” acted as a motivating factor for high altitude climbers to take part in risky expeditions. Similarly, Raue et al. [40] found that the combination of physical activity and feelings of control distorted the risk perception of indoor climbers. By contrast, fear is related to a lack of control and, therefore, increases the perceived risk [38].

#### 1.1.3. Emotions and Risk Exposure in Avalanche Terrain

Skiing in avalanche terrain is a complex, and physically and emotionally rewarding activity with the potential for high levels of excitement [20]. In avalanche terrain, people with bounded knowledge and information need to make complex decisions about terrain, snowpack, and group dynamics, often under time and weather constraints. They further must balance the risk of being caught in an avalanche to the reward of skiing powder in potentially dangerous terrain.

Other than the most obvious signs of instability, a mountainous snowpack is relatively opaque, and highly variable with respect to avalanche hazard. Furthermore, the snowpack also provides poor decision feedback; that is, we may not always receive immediate or accurate feedback to our decisions to ski selected terrain, and the absence of corrective feedback (i.e. an avalanche or other signs of instability) empowers continued, potentially incorrect decisions. This is often referred to as a “wicked” decision environment [49]. The “wickedness” of the learning environment adds to the complexity of the decision space. Decision making in this setting is more complicated than kind learning environments.

The most effective way to avoid avalanches is to avoid avalanche terrain altogether. From a recreational skiing perspective, however, avoidance is typically not realistic for most users because the more favorable ski terrain is often also potential avalanche terrain. Skiers have therefore adopted a set of behaviors to mitigate hazardous terrain. A typical backcountry ski tour would consist of a small group of enthusiasts traveling to a ski tour destination (e.g., a specific trailhead/parking area), after acquiring the detailed avalanche forecast (if available) from an avalanche forecast center in their region. They would then assess their tour plan and make their way to the ski destination—typically a snowfield or alpine summit from which they would determine a descent route based on snowpack stability. The route both up and down would be contingent on a complex skill set that includes an ability and willingness to hold an ongoing group discussion of weather, time, and distance constraints; risk assessment of terrain and snowpack; as well as an assessment of group expertise and level of risk aversion. In addition, all the members of the group would carry tools that aid in locating a buried avalanche victim. These include an avalanche transceiver, probe, and a shovel for extrication. The combination of planning and communication coupled with rescue equipment are minimal expectations of behavior among skiers, and the absence of these would be considered to increase the risk to an unacceptable level in most groups. Other high-risk factors include skiing alone in risky terrain under elevated hazard conditions or skiing very exposed terrain with severe consequences when the avalanche hazard is high.

In response to the relatively high number of fatalities caused by human decision errors, there has been an increased emphasis on understanding the role of decision making in avalanche terrain in recent years [2,11,50,51,52,53,54,55,56]. In the avalanche community, decision making, and the factors that influence these processes in avalanche terrain has broadly been termed the “human factor” [57]. The awareness of these human factors was greatly increased by the pivotal work by McCammon [58,59] where he attributed decision failures to social dynamics (i.e., gender mix, leadership, the presence of others) but did not specifically consider either the role of physical activity or emotional state on the decision-making process. His use of a post-mortem approach to fatal accident analysis could not determine the emotional state of skiers at the time of the accident. Understanding how emotions affect judgement and decision-making in complex environments, such as avalanche terrain, is important. If emotions have an effect, and if people fail to recognize this, they are likely to be caught by surprise both by their own and others’ behavior [9]. In a series of studies aimed at assessing the affective state of skiers with respect to risk, Stephensen and Matiny [60] presented backcountry skiers with a range of terrain scenarios and asked them to rate how much they liked it, and then how risky they found the descent. Across all the studies, backcountry skiers judged the scenarios they liked to be less risky. Tiedens and Linton [61] demonstrated that high certainty emotions such as happiness and anger lead to less deliberate thinking. Low certainty emotions such as sadness and fear lead to higher levels of deliberate reasoning.

### 1.2. Aims of the Study

This research has expanded the notion of human factors in avalanche accidents by employing methods from Ariely and Lowenstein [9] to understand how emotions, associated with powder fever, may or may not alter skier decision making in potentially dangerous backcountry circumstances. The aim of this paper was to answer the research question: “Do emotions, linked to powder fever in the backcountry, affect the assessment of, and the willingness to expose oneself or others to, take risks?”

**Figure 1:**
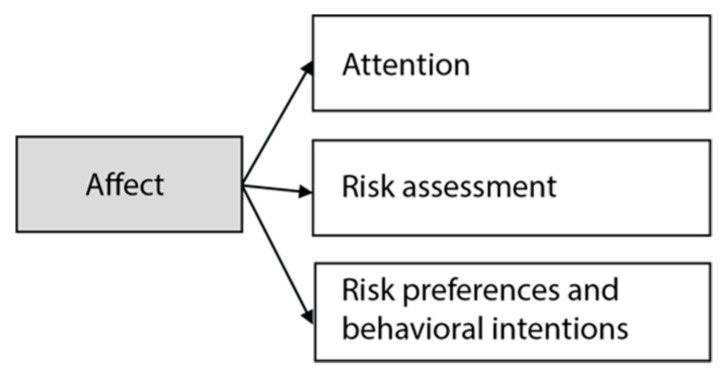
Theoretical framework.

Our theoretical framework is summarized in Figure 1, below.

Our work pursued two complementary lines of inquiry to evaluate how emotions affect risk perception, judgment, and behavior. One method used movies to induce positive and negative emotional states, and the other approach used the in-person experience of skiing powder. Our first two studies attempted to test the effect of emotions on attention to danger signs and risk judgements with visual visceral powder stimuli (ski movies). Specifically, we tested the role of media viewing on risk perception using both a positive and negative viewing experience. We tested for changes to both risk evaluation and personal risk perception resulting from respondents’ viewing experience.

In the third study, we emulated prior work by Ariely and Lowenstein [9] examining decision-making under sexual arousal, but rather than sexual arousal, we examined the effect of powder fever by means of real powder stimuli (skiing exciting terrain in good snow). Specifically, we wanted to examine if powder fever affects intentions to engage in risky behaviors in the specific setting of avalanche terrain. Across all three studies, we also wanted to examine and compare if the method of invoking the stimuli affects the magnitude and direction of the measured response.

## 2. Study 1

### 2.1. Materials and Method

#### 2.1.1. Hypotheses

**Hypotheses** **1** **(H1).** *Participants judge risk to be lower after seeing a positive ski film compared to participants seeing a negative ski movie.*

**Hypotheses** **2** **(H2).** *Attention to signs of danger (avalanche clues) is lower (higher) after seeing a positive (negative) ski movie.*

#### 2.1.2. Participants

We posted an invitation to participate in an experiment in Tromsø, Norway, on the CARE Facebook page (@careuit). Participants were told that they would see a movie and answer a short questionnaire, and that pizza would be served. Fifty-four subjects (16 female, 38 male) aged 20 to 48 years (M = 27.54, SD = 5.71) participated in the experiment on two separate evenings. Of these, 44 provided complete answers to all questions.

#### 2.1.3. Procedure

The experiment had the following three parts: A pre-survey, a movie treatment, and a post-survey. We sent out the pre-survey via email to all participants who signed up for the experiment. The pre-survey contained questions about backcountry skiing experience and skills, avalanche education, and risk preferences. The participants indicated their informed consent on the first page of the questionnaire. Only participants who filled out the pre-survey were invited to the movie experiment.

The movie experiment took place on two evenings, one to two days after the pre-survey. We screened a movie with positive emotional content on the first movie night, and a movie with negative emotional content on the second night. Both movies were approximately 15 min long. We allowed participants to choose which night to participate in the experiment. Participants knew that the movies would be about skiing but were unaware of the screening order.

During the movie experiment, participants were instructed to open, but not start the second survey (post-test) on their mobile phone before the movie started. They then watched the movie for 15 min. Immediately after the screening, participants were asked to advance to the first page of the survey, where they answered questions about their emotional response to the movie. The second section of the survey assessed participants’ ability to identify relevant informational cues. We sequentially showed a set of 12 photos (3 s each), and thereafter asked participants to identify which cues had and had not been shown. The method to ask participants to rate or evaluate photos of avalanche prone descents has been used in previous studies [56,60]. The last section of the survey focused on risk assessment. Participants sequentially saw 15 different pictures (7 s each) of backcountry ski runs. After a run was displayed, the participants were asked to evaluate how risky it would be for them to ride the run under similar conditions. The experiment ended when all 15 runs had been displayed and evaluated. The photos and risk scenarios were shown on a large screen placed in front of the participants in an auditorium (same as the ski movie). Consequently, the order of presentation was the same for all participants. The experiment was reviewed and approved by the Norwegian Center for Research Data prior to data collection (NSD 56569).

#### 2.1.4. Materials

The movie experiment had the following two treatments: positive and negative affect. We induced positive affect via a movie showing skiers successfully riding steep terrain in good snow, and negative affect via a movie showing skiers being involved in an avalanche accident (links to films and images used in this study are available through osf: https://osf.io/36dhf (accessed on 7 August 2021)). We chose to use ski films, since movies have been found to induce stronger arousal than pictures do [62].

We measured participants’ emotional reactions to the movie stimuli with two questions: (1) “We would like to reflect on your current feelings and answer what type of feelings this movie elicited” (scale: 1 “very negative emotions”, 4 “neither positive nor negative emotions”, 7 “very positive emotions”), and (2) “How much would you say you liked the movie?” (scale: 1 “not at all” to 7 “Very much”).

To evaluate if the movie stimuli influenced attention and information seeking, we used a series of 12 photos taken on backcountry trips. The pictures contained both relevant avalanche danger signs (e.g., fresh avalanches, wind-loaded snow) and irrelevant information (e.g., a snowboard, a helmet camera, see the Appendix A). Participants indicated what they had seen in the pictures on a list containing 8 relevant avalanche danger signs and 7 irrelevant information clues. We used the number of correctly identified danger signs as a measure of attention. We used the following two trait measures to evaluate potential differences in risk preferences between the two treatment groups: the “Brief Sensation-Seeking Scale” (BSSS) [63], and the “Stimulating-Instrumental Risk Index” (SIRI) [64].

The BSSS consists of the following four subscales: (1) experience seeking, (2) boredom susceptibility, (3) thrill and adventure seeking, and (4) disinhibition. Each subscale contains two statements, for a sum of eight statements in total. Examples include “I would like to explore strange places” (experience seeking), “I prefer friends who are excitingly unpredictable” (boredom susceptibility), “I like to do frightening things” (thrill and adventure seeking), and “I like wild parties” (disinhibition). The participants answered on a five-point Likert-like scale ranging from 1 “strongly disagree” to 5 “strongly agree”. The BSSS scale as a whole had a sufficiently high interim correlation (Cronbach’s α = 0.80) in our study. However, the items in the individual subscales displayed relatively low scale reliability coefficients (experience seeking: α = 0.45, boredom susceptibility: α = 0.55, thrill seeking: α = 0.62, disinhibition: α = 0.79). We, therefore, collapsed BSSS into one measure. SIRI consists of the following two subscales: (1) Stimulating risk taking (four statements), and (2) Instrumental risk taking (three statements). Example statements include “When I pursue my passions, I like the moments of balancing on the edge of risk” (stimulating risk-taking), and “I take the risk only when it is necessary to reach my goal” (instrumental risk-taking). All questions were measured on the same scale as the BSSS. The SIRI scale did not have a sufficiently high interim correlation in our study (Full scale: α = 0.55, Stimulating risk-taking: α = 0.52, Instrumental risk-taking: α = 0.49). We, therefore, refrained from using this measure.

Experience with backcountry skiing was measured with the following two questions: “During the past 5 riding seasons, how many days per season did you on average ride back/sidecountry?”; and “During how many seasons in total have you been an active (at least one trip per year) back/sidecountry rider?”. We measured riding skills with the question; “What is your level of skills would you say you have as a skier?” (scale: 1 “beginner” to 7 “Expert”). Finally, we asked participants to rate their avalanche training on a scale from 1 “No training” to 7 “Expert”. All survey questions are available in Table S1 in the supplementary material. 

### 2.2. Results

Forty-four participants signed up for the experiment and filled out the pre-survey. Of these, 21 saw a movie with negative emotional content (Group 1), and 23 saw a movie with positive emotional content (Group 2). Seventeen of the 21 participants in group one were male, while the corresponding number for group two was 15. We present descriptive statistics and tests for differences between the two samples in Table 1. The Shapiro–Wilk test suggested that some of our variables were non-normally distributed. We used Mann–Whitney U-tests to evaluate differences in means for these variables.

As can be seen in the last panel of Table 1, the differences in backcountry experience avalanche knowledge, or age between the two samples were not significant at the five percent level. However, the subjects in group one were significantly more sensation-seeking (BSSS) than the subjects in group two (t = 2.080, *p* = 0.044). This difference was not explained by the uneven gender balance in the two groups. When we ran a regression where we controlled for treatment and gender, we found that the treatment had a significant effect, while gender did not. When we included an interaction between the treatment and gender, all coefficients dropped below 5 percent significance. 

We present the results from the movie experiment in Table 2. The first two rows in Table 2 show the treatment effect on the reported emotions. Low numbers (1 to 3) represent negative emotions, while high numbers (5 to 7) represent positive emotions. Four represents neutral emotions. The participants in group one (negative affect), on average, reported mildly negative emotions, while the participants in group two (positive affect), on average, reported positive emotions. A *t*-test showed that the difference in reported emotions between the two groups was significant below the one percent level (t = −6.066, *p* < 0.001). 

#### 2.2.1. Results for Hypothesis H1

We evaluated the effect of positive and negative emotions on risk judgement by comparing the perceived risk of each of the 15 ski scenarios. We found no evidence that the participants who watched the positive ski movie evaluated the average risk differently from the participants that saw the negative ski movie (t = −0.094, *p* = 0.463). Only one of the 15 images (#14) was evaluated as less risky by the participants in the positive ski movie group (t = 2.619, *p* = 0.006). This effect became insignificant at a five percent level when we corrected for multiple testing (Bonferroni correction).

#### 2.2.2. Results for Hypothesis H2

We evaluated attention effects by comparing the number of correctly identified cues of heightened avalanche danger in the sequence with 12 images. We found no evidence that seeing a negative ski movie affected the number of correctly identified avalanche clues (t = −0.633, *p* = 0.265), or the number of remembered avalanche cues (correct or wrong) (t = −0.910, *p* = 0.184).

### 2.3. Discussion

The aim of study one was to test if positive and negative emotions, induced via a movie stimulus, influenced risk judgement and attention to danger signs. We predicted that positive (negative) emotions would reduce (increase) the perceived risk and reduce (increase) attention to danger signs. Our empirical analysis showed that, while the movies did affect the participants’ reported emotions, they did not affect risk judgement or attention to danger signs. We measured powder fever with one single question on positive and negative affect. This did not enable us to explore the wider emotional experience of different classes of positive emotions connected to powder fever. We, therefore, changed from a single-item measure of positive emotions to a ten-item emotional measure in the second study.

## 3. Study 2

The purpose of study two was to replicate the findings in study one on a larger sample, and to evaluate within-subject changes.

### 3.1. Materials and Method

#### 3.1.1. Hypotheses

**Hypotheses** **3** **(H3).** *Participants judge risk to be lower after seeing a positive ski film compared to participants seeing a negative ski movie.*

**Hypotheses** **4** **(H4).** *Participants are more willing to take ski-related risk after seeing a positive ski film compared to participants seeing a negative ski movie.*

#### 3.1.2. Participants

We carried out the experiment during two avalanche seminars in two towns in northern Norway. Participants attended the seminars to learn about risk mitigation and decision-making in avalanche terrain. In total, 191 individuals over the age of 18 attended the two seminars. Attendance was uneven on the two locations, in spite of a similar population size of the two towns. One hundred and forty-eight individuals attended the first seminar (positive ski movie), while 43 attended the second seminar. Of the participants willing to state their gender, 105 were male and 69 were female. Average age was 37 (min = 20, max = 62, S.D = 10.38). One-hundred and thirty-nine participants provided complete answers to all questions (108 in the positive treatment, and 31 in the negative treatment).

#### 3.1.3. Procedure

To prevent a priming effect on the results from the content of the avalanche seminar, the study was carried out in the beginning of the seminar, only preceded by a short welcome. The participants were asked to find their mobile phone and visit a link that led them to a questionnaire. They were told to answer the first part of the questionnaire including informed consent, a general willingness to take risk, assessment of current emotions, and their level of backcountry skiing experience, avalanche education, age, and gender. The film was then screened in the auditorium. Immediately after the film ended, the participants were asked to advance in the questionnaire to the second part of the questionnaire including the same assessment of current emotions. The participants were then presented with a scenario showing a photo of a ski descent and asked to imagine that they were standing at the top of the slope. The participants were provided with relevant risk information related to skiing the slope. The participants then indicated their perceived risk and willingness to ski this particular descent. The participants were then again presented with the question on how much risk they are willing to take when skiing. The experiment was reviewed and approved by the Norwegian Center for Research Data prior to data collection (NSD 56569).

#### 3.1.4. Materials

The movie stimuli used were the same as in study 1.

We measured the following nine different emotions before and after the movie stimuli: satisfaction, wellbeing, happiness, interest, engagement, focus, fear, anger, and sadness. The respondents answered the question “Feel how you feel right now. Please give a value for each of the emotions listed below” (scale: 1 = not at all, 7 = to a very high extent). There were only marginal differences between the different classes of positive emotions (hedonic emotions; pleasure, satisfaction, and happiness, and eudaimonic emotions; interest, engagement, enthusiasm, and immersion). The hedonic emotions returned a Cronbach’s α = 0.90 (pre) and α = 0.95 (post). The eudaimonic emotions returned a Cronbach’s α = 0.88 (pre) and α = 0.90 (post). We, therefore, create combined scores for items related to hedonic (“Happiness”) and eudaimonic (“Excitement”) emotions, respectively. The three variables theoretically measure distinct negative emotions, and we, therefore, kept them as separate measures.

Since neither the subscales of BSSS or SIRI had sufficient reliability coefficients in study 1, we chose to measure willingness to take risk with the simple question, “How willing are you to take risk when it comes to skiing” (scale: 1 = completely unwilling to take risk, 10 = very willing to take risk). This question has been shown to predict real-life risk-taking behavior relatively well [65]. As with questions on emotions, participants answered this question both pre- and post-treatment.

As in study 1, we also evaluated risk assessment in study 2. Due to the nature of the avalanche seminar, we could not evaluate a large set of different scenarios. Instead, we used a single scenario. Participants were shown a photo of a skier standing at the start of a ski run. The photo was from the perspective of the individual standing on the slope, i.e., Point of view (POV) and participants were asked to imagine that it was them standing on the top of the slope. We provided participants with relevant information about the risk associated with riding the slope. This information included inclination, temperature, geographical orientation of the slope, altitude, and an avalanche forecast describing the current snow conditions (see the Appendix A). Each participant answered the following three questions: 1) “How likely is it that you would ski this run?” (scale: 1 = very unlikely, 10 = very likely), 2), “How attractive is the run?” (scale: 1 = very unattractive, 10 = very attractive), and 3) “How risky do you think that it would be for you to ski the run, given current snow conditions?” (scale: 1 = very low risk, 10 = very high risk).

Finally, we asked participants about how many years and days per season they toured in avalanche terrain. Both these questions were measured on an interval scale (1= 0–5, 2 = 5–10 …, 6 = 30+). All survey questions are available in Table S2 in the supplementary material.

### 3.2. Results

We present the descriptive statistics for the two samples in Table 3, below. The participants in group one (negative affect) had significantly more avalanche training and skied more days in avalanche terrain than group two (positive affect). The participants in group one also expressed stronger hedonic emotions (happiness), anger, and sadness prior to the movie stimuli than the participants in group two.

The main results of study two are presented in Table 4 and Table 5 below. Table 4 shows the differences in response between the two groups (between-subject), while Table 5 shows the differences in emotions and risk preferences before and after the movie stimuli (within-subject). Due to the non-normality of most variables according to the Shapiro–Wilk test, we evaluated all the differences with the Mann–Whitney U test for between-subject comparisons, and with the Wilcoxon signed rank test for within-subject comparisons.

As in study one, we found that the movie stimuli had a significant impact on emotions. The participants in the positive affect treatment experienced significantly more positive emotions and less negative emotions after the stimuli. Since the participants in group one reported less happiness and more anger prior to the movie screening, it is possible that this result is partly caused by the differences between the two samples. However, our within-subject analysis (Table 5) confirmed that the participants in group one (negative affect) experienced a significant increase in negative emotions after the movie, while the participants in group two (positive affect) experienced a significant increase in positive emotions.

#### 3.2.1. Results for Hypothesis H3

We found no significant differences in risk perception between group one and group two.

#### 3.2.2. Results for Hypothesis H4

We found no differences in the willingness to ski a potentially risky run or to take risk while skiing between the two groups. Indeed, we found that the participants in both treatment groups were less willing to take risks after having seen the ski movie.

### 3.3. Discussion

The results of study two replicated the findings in study one. Ski movies thus appear effective in terms of inducing positive and negative states of affect. However, we found no support for the hypothesis that emotions affect risk judgements or willingness to take risk. Our finding that the participants in both groups were less willing to take risks after seeing either ski movie may point to participants being more engaged to the setting and critical about risk decisions.

There are two potential problems with study two. The first is that the affective stimuli were relatively weak. The participants saw a movie about an unknown rider and were asked to judge risk in hypothetical scenarios. Riders enjoying real powder may experience stronger emotions. Second, the use of avalanche seminars may have made participants more self-conscious, and more focused on avalanche safety.

## 4. Study 3

The aim of study three was to test if “real” powder fever influences willingness to engage in behaviors that expose oneself and others to heightened levels of risk.

### 4.1. Materials and Method

#### 4.1.1. Hypothesis

**Hypotheses** **5** **(H5).** *Willingness to expose oneself and others to heightened levels of risk is higher when a rider has just ridden or anticipates riding powder (hot state) than when at home (cold state).*

#### 4.1.2. Participants

We recruited participants at the base of the Schlasman’s lift at Bridger Bowl ski area in SW Montana, USA. The lift only serves extreme ski and avalanche terrain, with the ski area notifying all customers that this area has an “Increased risk of avalanches, has no hazard markings, no grooming, no marked trails, steep chutes which may end in unmarked cliffs, and no easy way down” [66]. An avalanche transceiver is required to access this lift, and a shovel, probe, and partner are strongly recommended—all are items that are also required for safe backcountry travel. While this area is still within the ski area boundary, and is not backcountry, skiers using the ski lift can easily access complex and non-mitigated avalanche terrain by hiking a short distance from the top of the lift.

In total, 285 participants answered the in-field survey. Of these, 192 riders provided complete answers and were over 18 years old. Sixty participants are female and 132 are male. Mean age in the sample was 30 (SD = 11.54, min = 18, max = 68). About 40 percent were students at Montana State University. Sixty-one participants completed a follow-up survey at home.

#### 4.1.3. Procedure

We asked skiers waiting to board the ski lift to complete a 2-page field survey. The survey was printed on waterproof paper that could be completed with gloved hands using a marker. On the first page of the survey, participants first answered questions about their emotional state, and thereafter answered questions about their willingness to engage in potentially risky backcountry activities. The second page of the survey contained questions about backcountry and avalanche skills, and basic demographics. Participants provided informed consent on a separate paper. The number of questions and layout of the survey were restricted by the fact that participants would complete the survey in the field at the base of the lift, while waiting for their next ski lift (approx. 2–10 min depending on crowds). Complete surveys were then placed into a survey “mailbox” at the front of the lift line, right before boarding the next available chairlift.

Within 14 days, we sent each participant a follow-up online survey with the same questions on emotional state and questions on risk taking in the backcountry. The online survey followed the same basic structure as the field-survey. Participants first answered questions about their emotional state, and thereafter indicated their willingness to engage in risky backcountry behavior. In the last sections of the online survey, we asked subjects about their risk preferences, and more detailed socio-demographic characteristics. The experiment was reviewed and approved by the MSU Institutional Review Board [JJ010919-EX] on 9 January 2019.

#### 4.1.4. Material

Our experiment design was inspired by the approach used by Loewenstein and Ariely (9), who first asked subjects to answer questions related to sexual risk taking in a cold state, and thereafter in a sexually aroused state.

We first collected data from participants, who could be expected to be in a “powder aroused” state, at the base of Schlasman’s ski lift. We thereafter asked the same questions when the subjects could be expected to be in a cold state (at home). To evaluate the effect of our arousal treatment, we used the following four emotional states: happiness, excitement (stoke), fear, and anxiousness (nervousness). Participants answered on a scale from 1 “Not at all” to 7 “To a very high extent”.

We measured willingness to engage in risky behavior with seven questions. These questions were based on the set of questions used by Loewenstein and Ariely [9] but adapted to a backcountry setting.

Skiing in extreme terrain, whether inbounds at a ski area or in the backcountry, is associated with a set of obligatory behaviors conducive to safe skiing. This includes all skiers carrying and knowing how to use an avalanche transceiver, a type of emergency locator beacon that transmits and receives a radio signal for the purpose of finding people buried under snow, an avalanche probe for physically locating the victim, and a shovel for extrication. Skiers are also encouraged to ski with a partner and avoid avalanche terrain under high hazard conditions. As part of learning the technical skills to ski in increasingly hazardous terrain, there is also an associated body of knowledge that people participating in this sport are expected to acquire. These behaviors are deeply ingrained in the culture of the sport. A couple of decades ago it would have been socially acceptable to ski without this equipment and knowledge, whereas contemporary skiers often carry the equipment and engage in avalanche awareness education as part of identifying as a backcountry skier [4,67].

The first four risk questions asked participants to what extent they could imagine engaging in behaviors that exposed themselves to heightened levels of risk. The last three questions asked participants to what extent they would engage in behaviors that potentially exposed others to risk. Participants answered on a scale from 1 “No” to 7 “Yes, absolutely”. Four was defined as “Possibly”. The full set of questions is presented in Table 6.

Questions about backcountry experience and avalanche experience and knowledge were the same as in study 1. All survey questions are available in Table S3 in the supplementary material.

### 4.2. Results

The attrition out of the sample may have been systematically correlated with factors that affect the results. To ensure that the selection into the sub-sample did not distort the results in an important way, we analyzed differences between participants who only took the Hot survey (N = 176), and participants who took both the Hot and the Cold survey (N = 62). We performed Mann–Whitney U tests on all the non-normally distributed variables. The results are presented in Table 7.

As can be seen in Table 7, there were no statistically significant differences in intentions to engage in risky behaviors between group one and group two (hot and cold). There was neither any differences in self-assessed backcountry skills, avalanche training, or percentage of students. The percentage of women was slightly higher in group two (60.4 percent) than in group one (67.7 percent, χ^2^ = 3.040, *p* = 0.081).

Table 8 presents descriptive statistics and test statistics (Wilcoxon signed rank tests) for hot and cold states. As can be seen in the table, participants, on average, felt significantly happier and more excited when they were out skiing than when they were at home. We found no significant differences in negative emotions.

#### Results for Hypothesis H5

We, in general, did not find significant differences in individual items measuring the willingness to engage in risky behavior. The exception is “willingness to ski with a partner with unknown backcountry travel skills” (z = 3.212, *p* = 0.001).

A factor analysis suggested that our questions on willingness to engage in behaviors that expose oneself or others to risk behavior loads on two factors corresponding to the “Can you imagine” (Cronbach’s α = 0.788) and “Would you” (Cronbach’s α = 0.787) questions. We, therefore, present the total scores on the two sets of questions, in addition to presenting results for individual items. While we did not find a significant difference on the sum of scores on the “Would you” questions, we did find a significant difference (z =2.085, *p* = 0.037) between the sum of scores on the “Can you imagine” questions in a hot and cold state.

### 4.3. Discussion

The aim of study three was to test if powder fever affects increase the willingness to engage in behaviors that potentially exposes oneself or others to risk. We found weak evidence that the participants were more willing to engage in behavior that exposes themselves to risk in the field than at home, but there was no support for the hypothesis that powder fever incentivized behavior that exposes others to risk.

A large percentage of our participants answered “No” (lowest possible answer) to many of our questions in the field. More than 70 percent answered no to questions related to wearing a beacon, skiing alone on a high avalanche danger day, and leaving a partner who says no. About 50 percent answered no to questions about skiing with a partner on a high avalanche danger day, feeling frustrated with a partner who refuses to ski, and persuading a partner who says no. This means that there was not much variation in the data between the hot and cold states. We reran our analysis on the sub-sample of participants who answered that they, at least to some extent, could imagine or would engage in the behaviors in a hot state. For this sub-sample, most differences were significant (see Table A1, in Appendix A). However, the sample sizes were very small, and the results may be biased by selection. It should also be noted that the sample obtained from the Schlasman’s lift at Bridger Bowl may not be representative of the wider backcountry community. All riders boarding the lift must wear beacons, the terrain is extreme, and the level of avalanche awareness in the community is relatively high. As such, our estimates may represent a lower bound, and participants from a less avalanche aware community may show more willingness to engage in risky behavior.

## 5. Limitations and Future Research

### 5.1. Limitations

Our study constitutes a first attempt to test how emotions affect behavioral intentions among riders in the backcountry community. As such, it has limitations. Perhaps most importantly, deliberational or theoretical choices are different from real-life behavior. It is, therefore, important to test if our findings hold for choices made in the backcountry. Second, our sample sizes were relatively small. This prevented us from analyzing the potential differences in the effects between different groups of backcountry riders. For example, Rydell et al. [10] found that group identification moderated the effect of anger on risk taking behavior. This effect may be present among backcountry riders as well, and the results from study three suggested that group differences are likely. Future studies should, therefore, try to reach a larger, and more heterogeneous (both in terms of socio-demographics and geographic location) sample of backcountry riders. Finally, powder fever is a well-known concept among backcountry riders, but it has no scientific definition.

Measuring emotions and its impact on risk behavior in backcountry skiing is challenging. Ideally, we would like to measure moment-to-moment emotions and their direct impact on objective risk exposure. However, measuring moment-to-moment emotions during skiing without them being affected by the measure itself is challenging. One way to do this is with face fronting cameras that capture facial expressed emotions. However, the current technology only records the participants’ level of happiness, which does not resemble the full catalog of positive emotions that one may experience in skiing (See [20] for a description of this method) Another way is to recreate the experience via methods such as the day-reconstruction method [68]. Further research should explore the efficacy of these methods.

It is also challenging to measure participants’ real behavior and what this means in terms of objective risk. Avalanche danger varies in space and time, and even with all the time and information available, it would be difficult to reach an objective measure of risk. We were, therefore, left with the option of directly asking the participants about their emotional experience and risk willingness or intentions in a post tense.

In our studies, we recorded self-reported emotions linked to the concept called “powder fever”. However, we have no way of knowing if the emotions were strong enough to induce a sense of high emotion. Hence, it may be that we did not arouse our participants enough to induce the intended behavior changes. Passively watching ski movies of anonymous skiers may not engage respondents’ emotions, even when participants are asked to imagine that it is them skiing. For future studies, it may prove fruitful to use methods including virtual reality cameras and ski-simulators, to induce a state of affect. To rule out that powder fever does not affect milder forms of risk-taking behavior, future studies should include scenarios with different levels of risk exposure.

In this paper, we have tried different ways of measuring participants’ risk assessment and willingness. In the first study, we used multi-item measures of risk assessment where we showed the participants several descents and asked them to evaluate the level of risk for each descent. In the second study, we provided the participants with one scenario accompanied with information about the terrain and snow conditions. Such single item measures have inherent limitations [69,70] and conclusions from such measures should be drawn with caution.

### 5.2. Conclusions

The aim of this paper was to analyze the role of emotions in behaviors that expose backcountry riders to avalanche risk. We conducted the following three experiments: two movie experiments in the laboratory and one field experiment. Our results showed that both seeing a ski movie and skiing affect riders’ emotional state. We operationalized powder fever as positive emotions. In the first study, we measured this with a single item, where the participants reported the level of positive and negative affect. In the second and third studies, we measured two different classes of positive emotions (hedonic and eudemonic) to see if this would give us a better understanding of the emotional profile of powder fever. Even though there are differences between these two types of positive emotions, they are rather small. Still, neither of our two first studies support the hypothesis that positive emotions affect risk assessment or risk taking. Our third study provided weak support that powder fever increases willingness to take risk. However, the majority of the sample found it completely unacceptable to engage in the suggested behaviors. These are reassuring findings, as it may indicate that backcountry experience and avalanche training prepare riders to resist temptations, and that well established concepts related to risk are well entrenched in the populations tested in these studies.

## Figures and Tables

**Table 1:** Descriptive statistics.

	Group 1: Negative Affect		Group 2: Positive Affect				
	M	S.D.	M	S.D.	Diff	Test-Statistic	*p*-Value
Age	26.81	6.52	27.52	5.88	−0.71	−0.897 ^a^	0.370
Percentage of males	0.81	0.40	0.65	0.49	0.16	1.370 ^b^	0.242
Ski days per season	24.14	16.41	17.35	17.85	6.80	1.746 ^a^	0.081
Years BC experience	7.76	7.80	6.87	5.86	0.89	0.154 ^a^	0.878
Avalanche training	3.14	1.28	3.09	1.76	0.06	0.120 ^c^	0.905
BC riding skills	4.52	1.40	4.48	1.44	0.05	0.106 ^c^	0.916
BSSS	3.63	0.71	3.16	0.78	0.47	2.080 ^c^	0.044
N obs	21		23				

^a^ Mann–Whitney U test. ^b^ Chi-square test. ^c^ Student *t*-test.

**Table 2:** Mean responses and bivariate tests of differences between negative and positive affect.

	Group 1: Negative Affect		Group 2: Positive Affect				
	M	S.D.	M	S.D.	Diff	Test-Statistic	*p*-Value
**Affect**							
Emotion	3.40	1.45	5.65	1.19	−2.41	−6.066	<0.001
Like	5.57	1.03	5.35	0.98	0.22	0.738	0.232
**Risk assessment**							
Scene 1	2.76	1.09	2.61	1.16	0.15	0.655	0.327
Scene 2	5.48	0.68	5.78	1.04	−0.31	−1.143	0.327
Scene 3	2.62	1.20	2.61	1.37	0.01	0.027	0.130
Scene 4	5.10	1.04	4.65	1.11	0.44	1.359	0.490
Scene 5	2.57	1.03	2.78	1.51	−0.21	−0.230 ^a^	0.818
Scene 6	5.05	1.12	5.17	1.27	−0.13	−0.349	0.297
Scene 7	2.29	1.10	2.61	1.16	−0.32	−0.946	0.364
Scene 8	3.57	1.16	3.87	1.14	−0.30	−0.857	0.198
Scene 9	3.71	1.27	3.87	1.66	−0.16	−0.346	0.366
Scene 10	4.14	0.91	3.83	1.34	0.32	0.910	0.184
Scene 11	3.90	1.41	4.09	1.08	−0.18	−0.483	0.316
Scene 12	6.05	1.12	6.26	0.96	−0.21	−0.571 ^a^	0.568
Scene 13	4.57	0.98	4.48	1.20	0.09	0.280	0.390
Scene 14	4.95	1.24	4.00	1.17	0.95	2.619	0.006
Scene 15	5.86	0.91	6.26	0.81	−0.40	−1.497 ^a^	0.134
Scene Total	62.62	9.30	62.87	8.43	−0.25	−0.094	0.463
**Attention**							
N correct signs	11.76	1.81	12.09	1.59	−0.33	−0.633	0.265
N danger signs	7.86	1.01	8.17	1.27	−0.32	−0.910	0.184
N obs	21		23				

^a^ Mann–Whitney U test.

**Table 3:** Descriptive statistics.

	Group 1: Negative Affect		Group 2: Positive Affect				
	M	S.D.	M	S.D.	Diff	Test-Statistic	*p*-Value
Age	35.55	10.74	37.50	10.24	−1.95	−0.975 ^a^	0.330
Male gender	0.32	0.48	0.43	0.50	−0.10	1.069 ^b^	0.301
Ski days per season	3.74	1.77	2.38	1.47	1.36	3.760 ^a^	<0.001
Years BC experience	2.10	1.33	1.91	1.35	0.19	1.055 ^a^	0.292
Avalanche training	2.65	1.36	1.90	1.16	0.75	2.951 ^a^	0.003
Pre-test risk preferences (Dohmen)	4.74	1.97	4.41	1.89	0.33	0.895 ^a^	0.371
Pre-test emotions							
Happiness	4.25	1.14	4.68	1.21	−0.43	−2.03 ^a^	0.042
Excitement	4.76	1.23	4.66	0.99	0.10	0.609 ^a^	0.542
Fear	2.23	1.73	1.60	1.08	0.62	1.708 ^a^	0.088
Anger	1.65	0.95	1.14	0.44	0.51	4.030 ^a^	<0.001
Sadness	1.97	1.47	1.31	0.69	0.65	3.086 ^a^	0.002
N obs	31		108				

^a^ Mann–Whitney U test. ^b^ Chi-Square test.

**Table 4:** Mean responses and bivariate tests of differences between negative and positive affect. Mann–Whitney U tests.

	Group 1: Negative Affect		Group 2: Positive Affect					
	M	S.D.	M	S.D.	Diff	Test Statistic	*p*-Value	Effect Size
**Affect**								
Happiness	2.94	1.18	5.28	1.11	−2.35	−7.206	<0.001	−2.09
Excitement	4.56	1.14	5.34	1.11	−0.78	−3.350	<0.001	−0.70
Fear	4.68	1.83	2.13	1.35	2.55	6.284	<0.001	1.73
Anger	2.58	1.5	1.26	0.75	1.32	6.766	<0.001	1.37
Sadness	3.23	2.06	1.39	0.86	1.84	5.595	<0.001	1.49
**Risk instruments**								
Risk perception	6.16	2.16	5.45	2.04	0.71	1.757	0.079	0.34
Willingness to ski	4.74	2.59	5.23	2.72	−0.49	−0.836	0.403	−0.18
Risk preferences	4.32	1.76	3.87	1.76	0.45	1.275	0.203	0.26
N obs	31		108					

**Table 5:** Within-subject comparisons. Wilcoxon signed rank tests.

	Pre-Test		Post-Test					
	M	S.D.	M	S.D.	Diff	z-Value	*p*-Value	Effect Size
**Negative ski film**								
Happiness	4.25	1.14	2.94	1.18	1.31	4.335	<0.001	1.13
Excitement	4.76	1.23	4.56	1.14	0.20	0.621	0.535	0.16
Fear	2.23	1.73	4.68	1.83	−2.45	−4.827	<0.001	−1.40
Anger	1.65	0.95	2.58	1.50	−0.94	−3.618	<0.001	−0.70
Sadness	1.97	1.47	3.23	2.06	−1.26	−3.644	<0.001	−0.73
Risk preferences	4.74	1.97	4.32	1.76	0.42	2.309	0.021	0.32
**Positive ski film**								
Happiness	4.68	1.21	5.28	1.11	−0.60	−5.977	<0.001	−0.62
Excitement	4.66	0.98	5.34	1.11	−0.68	−5.966	<0.001	−0.65
Fear	1.60	1.08	2.13	1.35	−0.53	−3.837	<0.001	−0.37
Anger	1.14	0.44	1.26	0.75	−0.12	−1.010	0.312	−0.15
Sadness	1.31	0.69	1.39	0.86	−0.07	−0.431	0.667	−0.09
Risk preferences	4.41	1.89	3.87	1.76	0.54	4.211	<0.001	0.44

**Table 6:** Measures of intentions to engage in risky behavior in avalanche terrain.

Can You Imagine Riding on Slopes That Could Avalanche…? (Risk for Self)	
1.	...if you forgot your avalanche beacon (but managed to get through the gate), when the avalanche danger is considerable ^1^?
2.	… with a partner, who forgot his/her avalanche beacon (but managed to get through the gate), when the avalanche hazard is considerable?
3.	...alone, with the appropriate avalanche gear when the avalanche danger is high?
4.	…with a partner, who’s backcountry travel skills are unknown to you, when the avalanche danger is considerable?
5.	…with a partner and the appropriate avalanche gear, including an avalanche backpack or Avalung, when the avalanche danger is high>
**Would you…?** (Risk for others)	
6.	… feel frustrated if your ski partner is scared and refuses to ski an out of bounds run that you want to ski?
7.	...try to persuade a ski partner to ski an out of bounds run that you want to ski, if he or she is hesitant to ski?
8.	…try to persuade a ski partner to ski an out of bounds run that you want to ski, if he or she is nervous and says “no”?
9.	… leave your partner out of bounds to go ski a run if he or she is nervous and says “no”?

^1^ The use of “considerable” has meaning to backcountry skiers. It is a term used in the North American Avalanche Danger scale to denote the level of risk of avalanche. The five-categories are Low, Moderate, Considerable, High, and Extreme. Most avalanche fatalities occur at Considerable danger.

**Table 7:** Descriptive statistics—Group 1 (Hot) and Group 2 (Hot and Cold).

	Group 1		Group 2				
Variable	Mean	S.D.	Mean	S.D.	Diff	Test Statistic	*p*-Value
**Socio-demographics**							
Age	28.82	11.13	31	12.33	−2.18	−1.222 ^a^	0.223
Percentage Male	0.72	0.45	0.62	0.49	0.09	1.734 ^b^	0.188
Student	0.40	0.49	0.44	0.50	−0.05	0.359 ^b^	0.549
Avalanche training	3.28	1.24	3.02	1.16	0.27	1.365 ^c^	0.172
BC skills	4.25	0.78	4.33	0.65	−0.08	−0.393 ^c^	0.695
**Emotions**							
Excited	6.02	1.14	5.87	0.92	0.15	1.426 ^c^	0.154
Happy	6.44	0.78	6.30	0.78	0.15	1.410 ^c^	0.159
Anxious	2.26	1.48	1.97	1.21	0.29	1.315 ^c^	0.188
Scared	1.66	1.26	1.44	0.92	0.22	1.324 ^c^	0.186
**Risk taking**							
*Can you imagine skiing a slope that could avalanche…*							
… without a beacon?	1.68	1.32	1.59	1.35	0.09	0.962 ^c^	0.336
… with a partner w/o a beacon?	1.54	1.10	1.48	1.18	0.07	0.848 ^c^	0.396
… with a partner w unknown skills?	2.31	1.51	2.48	1.53	−0.17	−0.916 ^c^	0.360
… alone on a high avi day?	1.75	1.53	1.39	1.04	0.35	1.500 ^c^	0.134
… with a partner on a high avi day?	2.70	1.84	2.31	1.68	0.39	1.496 ^c^	0.135
Total imagine	9.98	5.97	9.25	5.40	0.73	0.814 ^a^	0.417
*Would you?*							
Feel frustrated if a partner says no?	2.02	1.29	2.13	1.52	−0.12	−0.227 ^c^	0.821
Leave a partner who says no?	1.56	1.22	1.57	1.16	−0.02	−0.213 ^c^	0.832
Persuade a partner who says no?	1.87	1.31	1.79	1.16	0.08	−0.042 ^c^	0.967
Persuade a partner who hesitates?	2.21	1.32	2.18	1.40	0.03	0.365 ^c^	0.715
Total would	7.66	3.82	7.67	4.10	−0.02	−0.026 ^a^	0.979
N obs	131		61				

^a^ *t*-test, ^b^ Chi-square test, ^c^ Mann–Whitney test.

**Table 8:** Mean responses in hot and cold states (group 2). Wilcoxon signed rank tests.

	HOT		COLD					
	Mean	S.D.	Mean	S.D.	Diff.	z-Value	*p*-Value	Effect Size
**Emotions**								
Excited	5.87	0.92	3.72	1.47	2.15	6.415	<0.001	1.32
Happy	6.30	0.78	4.69	1.31	1.61	6.037	<0.001	1.11
Anxious	1.97	1.21	2.33	1.61	−0.36	1.103	0.270	−0.19
Scared	1.44	0.92	1.38	0.99	0.07	−0.864	0.388	0.05
**Risk taking and norm violations**								
*Can you imagine skiing a slope that could avalanche…*								
… without a beacon?	1.59	1.35	1.66	1.42	−0.07	−0.268	0.789	−0.04
… with a partner without a beacon?	1.48	1.18	1.31	0.79	0.16	0.826	0.409	0.16
… with a partner with unknown skills?	2.48	1.53	1.82	1.04	0.66	3.214	0.001	0.43
… alone on a high avi day?	1.39	1.04	1.21	0.52	0.18	0.651	0.694	0.17
… with a partner on a high avi day?	2.31	1.68	1.77	1.23	0.54	1.836	0.066	0.31
Total score Imagine	9.25	5.40	7.77	3.65	1.48	2.085	0.037	0.31
*Would you?*								
Feel frustrated if a partner says no?	2.13	1.52	2.11	1.32	0.02	−0.615	0.539	0.01
Leave a partner who says no?	1.57	1.16	1.54	1.16	0.03	0.711	0.477	0.04
Persuade a partner who says no?	1.79	1.16	1.75	1.12	0.03	0.274	0.784	0.05
Persuade a partner who hesitates?	2.18	1.40	2.26	1.42	−0.08	−0.589	0.556	−0.07
Total score Would	7.67	4.10	7.67	4.28	0.00	−0.249	0.804	0.00
N obs	61		61					

## Data Availability

Data, stimulus, and test material is available through osf: https://osf.io/36dhf (accessed on 17 August 2021).

## Supplementary Materials

The following are available online at https://osf.io/36dhf/, Table S1: Study 1 questions, Table S2: Study 2 questions, Table S3: Study 3 questions.

## Appendix A

**Table A1:** Mean responses for participants who did not answer “No” in the hot state. Wilcoxon signed rank tests.

		HOT		COLD				
	N	Mean	S.D.	Mean	S.D.	Diff.	z-Value	*p*-Value
*Can you imagine skiing a slope that could avalanche…*								
… without a beacon?	14	3.57	1.70	2.50	1.95	1.07	1.657	0.098
… with a partner without beacon?	13	3.23	1.64	2.15	1.34	1.08	1.962	0.050
… with a partner with unknown skills?	40	3.25	1.35	2.15	1.12	1.10	3.870	<0.001
… alone on a high avi day?	11	3.18	1.47	1.64	0.81	1.55	2.430	0.015
… with a partner on a high avi day?	31	3.58	1.50	2.32	1.47	1.26	2.943	0.003
*Would you?*								
Feel frustrated if a partner says no?	31	3.23	1.45	2.87	1.34	0.35	0.859	0.391
Leave a partner who says no?	17	3.06	1.34	2.41	1.58	0.65	2.177	0.030
Persuade a partner who says no?	29	2.66	1.17	2.34	1.32	0.31	2.152	0.031
Persuade a partner who hesitates?	36	3.00	1.29	2.92	1.44	0.08	0.273	0.785
